# What Is the Relationship between Chronotype and Disordered Eating in Adolescents? The EHDLA Study

**DOI:** 10.3390/nu16162576

**Published:** 2024-08-06

**Authors:** José Francisco López-Gil, Jorge Olivares-Arancibia, Rodrigo Yáñez-Sepúlveda, Mayra Fernanda Martínez-López

**Affiliations:** 1 One Health Research Group, Universidad de Las Américas, Quito 170124, Ecuador; 2 Department of Communication and Education, Universidad Loyola Andalucía, 41704 Seville, Spain; 3 AFySE Group, Research in Physical Activity and School Health, School of Physical Education, Faculty of Education, Universidad de Las Américas, Santiago 7500000, Chile; jolivares@udla.cl; 4 Faculty Education and Social Sciences, Universidad Andres Bello, Viña del Mar 2520000, Chile; rodrigo.yanez.s@unab.cl; 5 Cancer Research Group, Faculty of Medicine, Universidad de Las Américas, Quito 170124, Ecuador

**Keywords:** chronobiology, circadian rhythms, circadian preference, eveningness, morningness, eating behavior, eating disorders, youths

## Abstract

Background: Evidence assessing the relationship between chronotype and disordered eating in adolescents is scarce. The current study tried to evaluate the association between chronotype and disordered eating in a sample of Spanish adolescents. Methods: This secondary cross-sectional study analyzed data from the Eating Healthy and Daily Life Activities (EHDLA) study. The sample consisted of 703 adolescents (56.3% girls) aged between 12 and 17 years from the *Valle de Ricote* (Region of Murcia, Spain). Chronotype was assessed using the Morningness/Eveningness Scale in Children (MESC). Disordered eating was evaluated by two psychologists using the Sick, Control, One, Fat, and Food (SCOFF) questionnaire. Results: Adolescents with an eveningness chronotype showed a higher SCOFF score (estimated marginal mean [*M*] = 1.1; 95% confidence interval [CI] 0.7 to 1.5) in comparison with adolescents with a morningness chronotype (*M* = 0.7; 95% CI 0.5 to 0.8) (*p* = 0.010), as well as with those with an intermediate chronotype (*M* = 0.6; 95% CI 0.5 to 0.8) (*p* = 0.032). A higher predictive probability of having disordered eating was identified in adolescents with an eveningness chronotype (39.5%; 95% CI 22.8% to 59.1%), compared to adolescents with an intermediate chronotype (14.9%; 95% CI 10.8% to 20.1%) (*p* = 0.008) and with their counterparts with a morningness chronotype (16.9%; 95% CI 11.6% to 24.0%) (*p* = 0.021). Conclusions: This study reveals that adolescents with an eveningness chronotype are more likely to exhibit disordered eating behaviors compared to those with morningness or intermediate chronotypes. These findings highlight the importance of considering chronotype in adolescent health, particularly in developing targeted interventions to prevent eating disorders.

## 1. Introduction

Eating disorders, including bulimia nervosa, anorexia nervosa, and binge eating disorder, are a group of psychiatric conditions characterized by unusual eating patterns and severe health consequences [1]. According to the Diagnostic and Statistical Manual of Mental Disorders, Fifth Edition (DSM-5) and the Eleventh Revision of the International Statistical Classification of Diseases and Related Health Problems (ICD-11), these disorders pose a significant public health challenge, particularly in middle- and high-income countries, due to their rising prevalence among young people in the past 50 years [2,3]. Eating disorders are among the most dangerous mental health conditions, leading to substantial years of life lost and deaths globally [4,5]. The causes of these disorders are multifaceted and involve multiple risk factors, and the prevalence is particularly high among adolescents and young adults [6,7]. A systematic review of prevalence studies conducted between 1994 and 2013 found widely varying estimates for the lifetime prevalence of eating disorders, ranging from 1.0% to 22.7% for women and 0.3% to 0.6% for men [8]. Adolescence is a period of vulnerability, which underscores the importance of recognizing and identifying disordered eating behaviors [9]. Unfortunately, many cases go undiagnosed and untreated due to stigma and shame [10,11]. In addition to eating disorders, there are also disordered eating behaviors that encompass actions like restrictive dieting, binge eating, and purging that do not meet clinical eating disorder criteria but may still impact health outcomes. Although distinct from diagnosed eating disorders, these behaviors are significant predictors of future eating disorder development and obesity in adolescents [12,13,14]. Given that the global prevalence of disordered eating is high [15], knowing the possible associated factors could be useful for its prevention. In Spain, previous research has reported that the prevalence of adolescents exhibiting disordered eating behaviors, as assessed by the Sick, Control, One, Fat, and Food (SCOFF) questionnaire, ranges between 12% and 30.1% [16,17,18].

Chronotype is one of several behavioral, sociodemographic, cultural, religious, and clinical factors that can affect eating behavior, as shown by research [19]. Chronotype refers to an individual’s natural tendency to sleep and wake up at a specific time of day, with variations ranging from morningness to eveningness [20]. Researchers have previously suggested that there is a continuum of chronotypes, which includes morningness and eveningness [21]. People who prefer to go to bed and wake up early and are most productive in the morning hours are classified as having morningness, while those who prefer to stay up late and are most productive in the evening hours are categorized as having eveningness [22]. Individuals who fall in between these categories are considered to have an intermediate chronotype. Studies have shown that most individuals exhibit greater morningness during childhood and then shift toward eveningness during adolescence, with some reverting back to morningness in late adolescence and early adulthood [23,24]. This shift has also been observed in longitudinal studies, such as the one conducted by Karan et al. [25], which pointed out that eveningness increases from age 14 to 19 before shifting toward morningness in young adulthood.

The relationship between chronotype and health is now being explored in greater depth [26]. Individuals with an eveningness chronotype are more prone to health problems and have a higher mortality compared to those with a morningness chronotype [27,28]. The identification of various clinical conditions linked to the eveningness chronotype suggests that chronotype should be considered an important factor in improving lifestyle and avoiding noncommunicable diseases related to an eveningness chronotype [29], such as cancer [30], type 2 diabetes [30], nonalcoholic fatty liver disease [31], and obesity [32]. Supporting this notion, a scoping review indicated that an evening chronotype is associated with later mealtimes, higher energy intake, especially before bedtime, skipping breakfast, and an unhealthy dietary pattern [33]. Additionally, an evening chronotype is linked to higher alcohol consumption [34] and poor diet quality, such as low adherence to the Mediterranean diet [35]. Recent studies also suggest that the evening chronotype is a risk factor for substance use and depressive disorders [36,37], while the morning chronotype serves as a protective factor [37]. Moreover, the evening chronotype is associated with anxiety symptoms, sleep disturbances, and poor lifestyle habits, which may elevate the risk for psychiatric disorders [36]. Despite this evidence, most research has focused on adults, with limited data available for young people [26,35].

More specifically, the association of chronotype with eating disorders is not entirely clear. For example, Esin et al. [38] found that chronotype was linked to emotional eating, anxiety, stress, depression, and life satisfaction, but not to body mass index and disordered eating among Turkish university students. Amicis et al. [39] noted that an evening chronotype was related to a greater prevalence of binge eating and/or food addiction among adults with overweight/obesity in Italy, though this was only significant in males. Romo-Nava et al. [40] identified that an evening chronotype correlated with higher mean Eating Disorder Diagnostic Scale (EDDS) composite z-scores and a greater prevalence of bulimia nervosa, binge-eating behavior, and nocturnal eating binges among US adults with bipolar disorder. In children and adolescents, an evening chronotype has been linked to a higher body mass index, increased fast-food consumption, and a greater likelihood of experiencing certain disordered eating behaviors, such as night eating syndrome and food addiction [26]. Moreover, there is a gap in the scientific literature regarding the relationship between chronotype and eating disorders in the adolescent population [26]. To our knowledge, no previous study has assessed the association between chronotype and disordered eating among adolescents. Thus, the aim of the current study was to evaluate the relationship between chronotype and disordered eating among Spanish adolescents.

## 2. Materials and Methods

### 2.1. Study Design and Population

This cross-sectional study examined participants aged 12 to 17 years old who were enrolled in the Eating Healthy and Daily Life Activities (EHDLA) study. The EHDLA study collected information from a representative sample of participants from the *Valle de Ricote* (Region of Murcia, Spain) in 2021 and 2022. The data were collected from three secondary schools, and the complete methodology of the study is available in another source [41]. The school timetable in *Valle de Ricote* is fairly uniform across the schools included in this study. Classes commence at 8 a.m., conclude at 2 p.m., and feature a 30 min break at around 11 a.m. In this study, we only involved a secondary sample with participants who had complete information on the variables of interest. Out of the initial 1378 adolescents, 523 were eliminated due to missing data on the SCOFF questionnaire, and 102 were removed due to missing data on chronotype. Additionally, 38 were excluded due to missing data on body mass index and 12 were removed due to missing data on energy intake. A final sample of 703 adolescents (56.3% of whom were girls) was included in the analysis.

The criteria for participation in this study were as follows: (1) being between the ages of 12 and 17, (2) residing in or registered with *Valle de Ricote*, and (3) providing consent from parents or legal guardians as well as assent from the student. Participants were excluded if they were exempt from physical education classes, had a medical condition requiring special attention, were contraindicated for physical activity, or were under pharmacological treatment due to a chronic medical condition.

This study received approval from the Ethics Committee of the Albacete University Hospital Complex, Albacete Integrated Care Management (ID 2021-85), and the Bioethics Committee of the University of Murcia (ID 2218/2018). The study was conducted in accordance with the principles outlined in the Declaration of Helsinki, and the human rights of the participants were respected.

### 2.2. Study Variables

#### 2.2.1. Chronotype (Independent Variable)

Adolescents’ chronotype was assessed using the validated Spanish translation by Díaz-Morales et al. [42] of the Morningness/Eveningness Scale in Children (MESC) [43]. The MESC is used to evaluate adolescents’ chronotype by asking questions about their preferences for morning or evening activities and their ability to perform tasks at specific times of the day. The reliability of the MESC as reported was supported by a Cronbach’s alpha of 0.82 [44]. This indicates a good level of internal consistency for the Spanish translation of the MESC. The MESC comprises 10 items with 4 or 5 possible answers for each, and a hypothetical scenario is presented to the adolescents, who indicate the statement that best identifies them, resulting in a score ranging from 1–4 (for seven questions) or 1–5 points (for three questions) (e.g., “How alert are you in the first half hour you’re up?” with answer options: (a) out of it; (b) a little dazed; (c) okay; and (d) ready to take on the world). Scores ranging from 10 (eveningness) to 43 (morningness) are possible, with lower scores indicating a preference for eveningness. Cutoff points of 18 and 30 points are typically used to classify morning types (≥30 points), intermediate types (19–29 points), and evening types (≤18 points) [43].

#### 2.2.2. Disordered Eating (Dependent Variable)

The SCOFF questionnaire, a self-report tool including five yes/no questions, is used to assess disordered eating. The Spanish version of the SCOFF, validated for primary care use [45], sets the cutoff at two positive answers out of five. One study including Spanish adolescents showed a specificity of 78% (95% CI 75% to 80%) and a sensitivity of 73% (95% CI 63% to 83%) for identifying eating disorders [46].

### 2.3. Covariates

Participants reported their age and sex, while socioeconomic status was evaluated using the Family Affluence Scale (FAS-III) [47], which calculates a score based on responses to six questions about family possessions. The FAS-III score ranges from 0 to 13 points, with higher scores indicating greater socioeconomic status. Adolescents’ height and weight were measured using standardized procedures [41], and their body mass index was calculated by dividing their weight in kilograms by their height in meters squared. Physical activity and sedentary behavior were assessed using the Spanish version of the YAP (YAP-S) [48], a 7-day recall questionnaire containing 15 items. This questionnaire uses a 5-point Likert scale divided into three domains: out-of-school activities, school activities, and sedentary habits. Physical activity and sedentary behavior scores were computed by adding the items in each domain. Adherence to the Mediterranean diet was assessed using the Mediterranean Diet Quality Index for Children and Teenagers (KIDMED) [49], and energy intake was estimated using a self-administered food frequency questionnaire (FFQ) that comprises 45 items and has been validated for use in the Spanish population [50].

### 2.4. Statistical Analysis

To assess the normal distribution of variables, density and quantile-quantile plots were used, along with the Shapiro–Wilk test. Categorical data are presented as counts (*n*) and percentages (%), while continuous data are presented as medians and interquartile ranges (IQRs). Since there was no significant interaction between chronotype and sex regarding SCOFF score or disordered eating (*p* > 0.05 for all), both girls and boys were analyzed together. To test the relationships between chronotype (i.e., “eveningness”, “intermediate”, or “morningness”) and SCOFF score or disordered eating among adolescents, generalized linear regression models (GLMs) were utilized. These models were used to address heteroscedasticity and outliers using robust methods [51]. For continuous outcomes, GLMs with a Gaussian distribution using the “*SMDM*” method were applied (i.e., using an S-estimate, an M-estimate, a design adaptive scale estimate, and another M-step). Additionally, the estimated marginal means (*M*) of the SCOFF score or predictive probabilities (%) of having disordered eating, along with their 95% confidence intervals (CI), were calculated based on the different chronotypes. All models were adjusted for several covariates, including sex, age, socioeconomic status, physical activity, sedentary behavior, adherence to the Mediterranean diet, and energy intake. Statistical analyses were carried out using R statistical software (version 4.4.0) by the R Core Team in Vienna, Austria, and RStudio (2024.04.1+748) from Posit in Boston, MA, USA. A *p*-value below 0.05 was selected as statistically significance.

## 3. Results

Table 1 depicts the descriptive data of the study participants according to their chronotype. The highest SCOFF median was observed in adolescents with an eveningness chronotype (median = 2.0; IQR = 3.0). Conversely, the lowest SCOFF median was identified in adolescents with morningness and intermediate chronotypes (median = 1.0; IQR = 2.0 for both). Furthermore, adolescents with an eveningness chronotype showed the highest proportion of disordered eating (56.4%), while the lowest proportion was found in adolescents with an intermediate chronotype.

**Figure 1:**
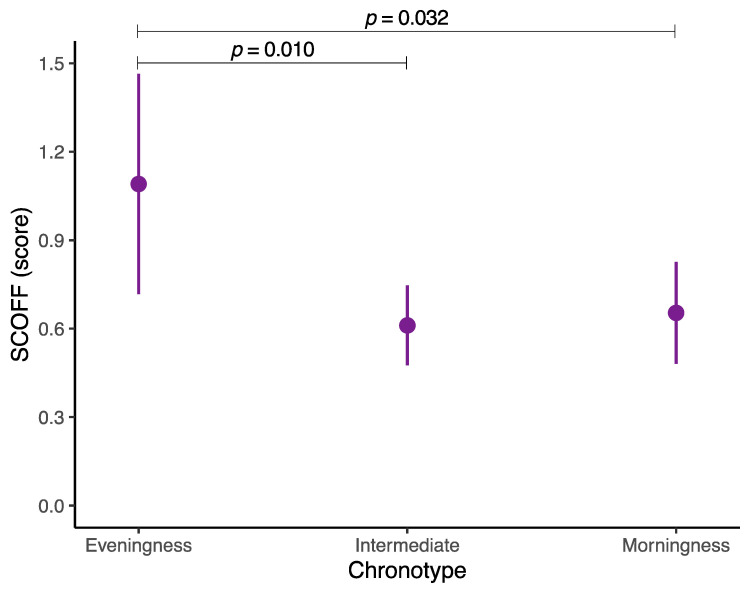
Estimated marginal means of Sick, Control, One, Fat, and Food (SCOFF) questionnaire based on the different chronotypes in Spanish adolescents. Data are shown as dots (estimated marginal means) and lines (95% confidence intervals). Adjusted for sex, age, socioeconomic status, physical activity, sedentary behavior, adherence to the Mediterranean diet, and energy intake. *p*-values for the difference in estimated marginal means among the different chronotypes were obtained from a Wald test for pairwise comparisons following a robust generalized linear model.

Estimated marginal means of SCOFF based on the different chronotypes are found in Figure 1. After adjusting for potential covariates (i.e., sex, age, socioeconomic status, body mass index, physical activity, sedentary behavior, adherence to the Mediterranean diet, and energy intake), we identified significant differences in the SCOFF score among the different chronotypes (i.e., eveningness, intermediate, and morningness chronotypes). Participants with an eveningness chronotype showed a higher SCOFF score (*M* = 1.1; 95% CI 0.7 to 1.5) in comparison with adolescents with a morningness chronotype (*M* = 0.7; 95% CI 0.5 to 0.8) (*p* = 0.010), as well as with those with an intermediate chronotype (*M* = 0.6; 95% CI 0.5 to 0.8) (*p* = 0.032). The full result of the GLM assessing the relationship between chronotype and SCOFF can be found in Table S1.

**Figure 2:**
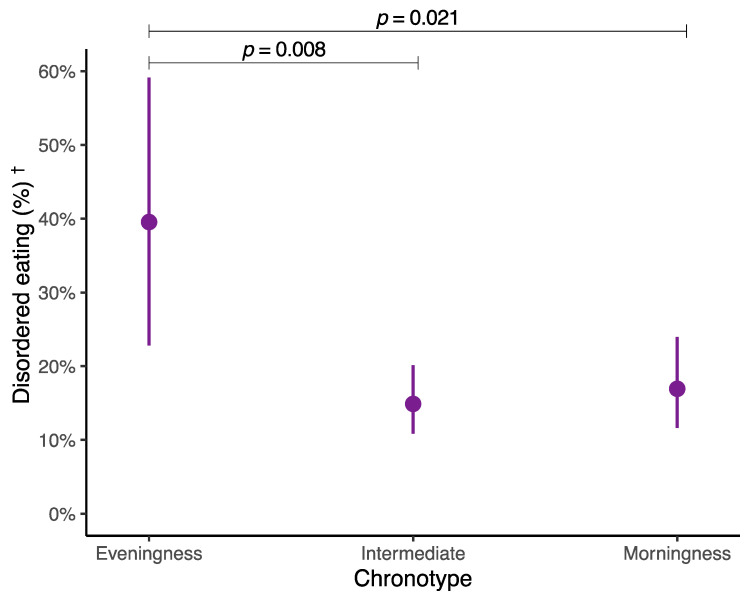
Predictive probabilities of disordered eating based on the different chronotypes in Spanish adolescents. Data are shown as dots (predictive probabilities) and lines (95% confidence intervals). Adjusted for sex, age, socioeconomic status, physical activity, sedentary behavior, adherence to the Mediterranean diet, and energy intake. ^†^ Cutoff point for disordered eating ≥ 2 points on the SCOFF questionnaire. *p*-values for the difference in predicted probabilities among the different chronotypes were obtained from a Wald test for pairwise comparisons following a robust generalized linear model.

Figure 2 indicates the predictive probabilities of experiencing disordered eating based on the different chronotypes. A higher predictive probability of having disordered eating was identified in adolescents with an eveningness chronotype (39.5%; 95% CI 22.8% to 59.1%), compared to adolescents with an intermediate chronotype (14.9%; 95% CI 10.8% to 20.1%) (*p* = 0.008) and with their counterparts with a morningness chronotype (16.9%; 95% CI 11.6% to 24.0%) (*p* = 0.021). These results were also adjusted for the above-mentioned covariates. Furthermore, Table S2 contains the complete results of the GLM analysis examining the relationship between chronotype and disordered eating.

## 4. Discussion

There is limited evidence evaluating the connection between chronotype eating disorders in adolescents. Only a previous study by Riccobono et al. [52] identified a negative correlation between the Morningness/Eveningness Questionnaire (MEC) and the Night Eating Questionnaire (NEQ), which means that the higher the score on the MEC (i.e., more nocturnal chronotype), the higher the score on the questionnaire assessing night eating syndrome). The night eating syndrome was included for the first time in DSM-5 as an “Other Specified Feeding or Eating Disorder (OSFED)”. Therefore, to our knowledge, this is the first study providing cross-sectional evidence about the relationship of chronotype with disordered eating among adolescents. Overall, these findings suggested that an evening chronotype was associated with disordered eating in the sample of Spanish adolescents examined. More specifically, adolescents with an eveningness chronotype showed lower estimated marginal means in the SCOFF and predictive probabilities of having disordered eating in comparison with their peers with an intermediate or morningness chronotype. This result is in line with earlier research, which suggested an association between an individual’s circadian rhythm, or chronotype, and their propensity towards disordered eating behaviors [36,53,54]. Although the specific mechanisms by which chronotype may promote the development of eating disorders are not known, it is important to know the specific mechanisms by which chronotype may promote the development of eating disorders or disordered eating, certain underlying mechanisms or influence factors have been described in the literature, such as clock genes, neuroendocrinology, the light/dark cycle, brain characteristics, sleep disorders psychological factors, and social factors [37]. Despite this fact, there are some possible reasons that could explain these findings.

On the one hand, adolescents are especially vulnerable to experiencing social jetlag [55,56]. This misalignment can lead to increased stress and difficulties in coping with daily life demands, affecting emotional regulation [55]. The chronic stress associated with social jetlag can lead to emotional distress and mental health issues such as anxiety and depression [57]. Adolescents might turn to disordered eating behaviors as a maladaptive coping mechanism to deal with negative emotions [58]. Emotional eating or binge eating episodes might temporarily alleviate stress [59] but ultimately contribute to feelings of guilt and shame [60], perpetuating a cycle of disordered eating and poor mental health. Additionally, evening chronotypes might have less time for social interactions and physical activities [61,62], which are important for maintaining a healthy lifestyle and positive body image, further increasing the risk of developing disordered eating behaviors.

Additionally, disruption of circadian rhythms and hormonal imbalance could also explain (at least partially) these results. Adolescents with an evening chronotype often have misaligned circadian rhythms due to late sleep onset and wake times, especially when combined with early school schedules [63]. This misalignment can disrupt the normal secretion patterns of hormones such as melatonin, cortisol, leptin, and ghrelin, which are crucial for regulating appetite and metabolism [64]. Leptin is known as a satiety hormone, and ghrelin is known as a hunger hormone [65]. A disrupted circadian rhythm can lead to increased ghrelin and decreased leptin levels, which can cause increased hunger and cravings, often for high-calorie, sugary, or fatty foods [66]. This hormonal imbalance may contribute to irregular eating patterns, binge eating, and other disordered eating behaviors [67]. Additionally, higher cortisol levels due to stress from circadian misalignment can lead to increased appetite and fat storage [68], further complicating the adolescent’s relationship with food.

Furthermore, evening chronotypes are more prone to experiencing sleep deprivation due to their natural preference for late-night activities [69], which conflicts with early morning obligations like school. Chronic sleep deprivation can impair cognitive functioning, including areas related to impulse control and decision-making [70]. Impaired cognitive functioning can lead to poor decision-making and impulse control regarding food intake [71]. Adolescents might be more prone to emotional eating, binge eating, or making unhealthy food choices when sleep-deprived [72]. Moreover, sleep deprivation affects the brain’s reward system, making high-calorie foods more appealing and harder to resist [73], thus increasing the likelihood of engaging in disordered eating behaviors. The lack of restorative sleep also contributes to fatigue, which can reduce motivation for physical activity, further exacerbating issues related to weight and body image [74].

On the other hand, chronotype appears to significantly influence individuals towards adopting an unhealthy dietary pattern, as reported by previous research across various age groups [35,75,76]. Specifically, earlier studies have identified that youths with an evening chronotype tend to show poorer diet quality compared to those with a morning chronotype [77,78]. An unhealthy diet often lacks the essential nutrients that the body needs to function properly. Nutrient deficiencies can affect mood, energy levels, and concentration, contributing to disordered eating behaviors [79]. Following a strict and unhealthy diet can foster a mindset of restriction, where food is seen as something to be strictly controlled [80]. This mindset can lead to a cycle of restriction and overcompensation (such as binge eating), which is characteristic of many eating disorders [81]. In addition, meal skipping, such as skipping breakfast, is more common among those with an eveningness chronotype in comparison with those with a morningness chronotype [72], and this behavior could mediate the association between chronotype and disordered eating [82]. Moreover, skipping meals could lead to less desirable psychological outcomes [83,84], including stress, anxiety, depression, or psychosocial health problems. These psychological factors are closely linked to the onset of eating disorders [85].

The findings from this study should be interpreted carefully due to several limitations. Firstly, as a cross-sectional study, it does not allow for causal conclusions. Furthermore, it is not possible to establish the direction of the association. Longitudinal studies are required to confirm whether a morning chronotype leads to higher levels of disordered eating in adolescents. Additionally, the data on chronotype and disordered eating were collected through questionnaires, which may introduce desirability bias and recall bias. However, these limitations are common with the use of questionnaires. Conversely, the study also has strengths, such as adjusting for multiple covariates in the analysis to reduce confounding effects and enhance the validity of the relationship between chronotype and disordered eating. Moreover, validated methods were used to assess both chronotype and disordered eating in the adolescent sample, and robust data analysis techniques were employed.

## 5. Conclusions

This study reveals that adolescents with an eveningness chronotype are more likely to exhibit disordered eating behaviors compared to those with morningness or intermediate chronotypes. The association persists even after accounting for various confounding factors such as sociodemographic, anthropometric, and lifestyle habits. These findings highlight the importance of considering chronotype in adolescent health, particularly in developing targeted interventions to prevent and treat eating disorders. Developing targeted interventions based on the findings involves tailoring strategies to fit the natural sleep–wake patterns of adolescents with an eveningness chronotype, such as flexible school schedules, healthy eating routines, increased morning light exposure, and providing mental health support. These interventions aim to reduce the risk of disordered eating behaviors by aligning with the adolescents’ chronotype and promoting overall health.

## Figures and Tables

**Table 1:** Descriptive data of the study participants according to physical literacy status (N = 703).

		Chronotype		
Variable		Eveningness	Intermediate	Morningness
Participants	*n* (%)	39 (5.5)	483 (68.7)	181 (25.7)
Sex	Boys (%)	13 (33.3)	194 (40.2)	100 (55.2)
	Girls (%)	26 (66.7)	289 (59.8)	81 (44.8)
Age (years)	Median (IQR)	13.0 (1.5)	14.0 (2.0)	13.0 (3.0)
FAS-III (score)	Median (IQR)	8.0 (3.0)	8.0 (2.5)	8.0 (3.0)
YAP-S physical activity (score)	Median (IQR)	2.4 (0.8)	2.6 (0.8)	2.8 (0.9)
YAP-S sedentary behaviors (score)	Median (IQR)	3.2 (0.8)	2.6 (0.8)	2.4 (0.8)
Body mass index (kg/m^2^)	Median (IQR)	22.1 (6.6)	21.6 (5.6)	21.7 (6.8)
KIDMED (score)	Median (IQR)	4.0 (3.0)	6.0 (3.0)	8.0 (3.0)
Energy intake (kcal)	Median (IQR)	2929.0 (1540.2)	2590.9 (1458.7)	2448.2 (1290.7)
SCOFF (score)	Median (IQR)	2.0 (3.0)	1.0 (2.0)	1.0 (2.0)
Disordered eating (%) ^†^	No (%)	17 (43.6)	345 (71.4)	128 (70.7)
	Yes (%)	22 (56.4)	138 (28.6)	53 (29.3)

FAS-III, Family Affluence Scale-III; IQR, interquartile range; KIDMED, Mediterranean Diet Quality Index for children and adolescents; SCOFF, Sick, Control, One, Fat, and Food; YAP-S, Spanish Youth Active Profile. ^†^ Cutoff point for disordered eating ≥ 2 points on the SCOFF questionnaire.

## Data Availability

The data used in this study are available upon request from the corresponding authors. However, given that the participants are minors, privacy and confidentiality must be respected.

## Supplementary Materials

The following supporting information can be downloaded at: https://www.mdpi.com/article/10.3390/nu16162576/s1, Table S1: Association between chronotype and Sick, Control, One, Fat and Food (SCOFF) score among Spanish adolescents; Table S2: Association between chronotype and disordered eating ^†^ among Spanish adolescents.
