# Supplementary material for: What Is the Relationship between Chronotype and Disordered Eating in Adolescents? The EHDLA Study
Source: Nutrients. 2024 Aug 6;16(16):2576. doi: 10.3390/nu16162576 (PMC11357602; doi:10.3390/nu16162576)
Supplement: Supplementary file 1 [file nutrients-16-02576-s001.zip › nutrients-3133098-supplementary.pdf]

Supplementary material

Table S1. Association between chronotype and Sick, Control, One, Fat and Food (SCOFF) score among Spanish adolescents.

Predictor	*B*	SE	LLCI	ULCI	*p*-value
Independent variable					
Eveningness	Reference				
Intermediate	-0.37	0.18	-0.72	-0.02	0.040
Morningness	-0.41	0.20	-0.80	-0.02	0.037
Covariates					
Age	-0.01	0.03	-0.06	0.05	0.808
Boys	Reference				
Girls	0.44	0.09	0.28	0.61	<0.001
FAS-III (per one point)	-0.05	0.02	-0.09	-0.01	0.017
YAP-S physical activity (per one point)	0.11	0.06	-0.01	0.24	0.084
YAP-S sedentary behaviors (per one point)	0.10	0.07	-0.04	0.25	0.161
KIDMED (per one point)	0.01	0.02	-0.02	0.05	0.414
Energy intake (per 1000 kcal)	0.00	0.02	-0.05	0.04	0.842

B, unstandardized beta coefficient; LLCI, lower limit confidence interval; SE, standard error; ULCI, upper limit confidence interval.

Table S2. Association between chronotype and disordered eating † among Spanish adolescents.

Predictor	OR	SE	LLCI	ULCI	*p*-value
Independent variable					
Eveningness	Reference				
Intermediate	0.42	0.34	0.22	0.82	0.010
Morningness	0.45	0.38	0.21	0.94	0.034
Covariates					
Age	1.05	0.06	0.94	1.17	0.380
Boys	Reference				
Girls	2.45	0.18	1.71	3.52	<0.001
FAS-III (per one point)	0.92	0.04	0.85	0.99	0.029
YAP-S physical activity (per one point)	1.26	0.13	0.97	1.64	0.081
YAP-S sedentary behaviors (per one point)	1.10	0.15	0.82	1.48	0.535
KIDMED (per one point)	1.03	0.04	0.96	1.11	0.373
Energy intake (per 1000 kcal)	1.00	0.04	0.92	1.08	0.929

B, unstandardized beta coefficient; LLCI, lower limit confidence interval; SE, standard error; ULCI, upper limit confidence interval. † Cutoff point for eating disorders ≥ 2 points on the Sick, Control, One, Fat and Food (SCOFF) questionnaire.
